# The Effect of Increased Sodium Intake with a Carbohydrate-Rich Meal on Glucose Homeostasis in People without Diabetes after Roux-en-Y Gastric Bypass: a Proof-of-Concept, Randomized, Open-Label, Crossover Study

**DOI:** 10.1007/s11695-025-08420-4

**Published:** 2025-11-28

**Authors:** Stanislava Katsarova–Harrison, Gráinne Whelehan, Joseph Henson, Louisa Y. Herring, David Bowrey, Alice E. Thackray, David J. Stensel, Iskander Idris, Oluwaseun Anyiam, Anjali Zalin, Alan Askari, David R. Webb, Tom Yates, Melanie J. Davies, Dimitris Papamargaritis

**Affiliations:** 1 Diabetes Research Centre, University of Leicester College of Life Sciences, Leicester, UK; 2 National Centre for Sport and Exercise Medicine, School of Sport, Exercise and Health Sciences, Loughborough University, Loughborough, UK; 3 Leicester Diabetes Centre, Leicester General Hospital, Leicester, UK; 4 Department of Surgery, University Hospitals of Leicester NHS Trust, Leicester General Hospital, Leicester, UK; 5 Faculty of Sport Sciences, Waseda University, Tokyo, Japan; 6 East-Midlands Bariatric & Metabolic Institute, University Hospital of Derby and Burton Foundation Trust, Derby, UK; 7 Bedfordshire Hospitals NHS Foundation Trust, Luton, UK; 8 Department of Diabetes and Endocrinology, Kettering General Hospital NHS Foundation Trust, Kettering, UK

**Keywords:** Roux-en-Y gastric bypass, Salt, Sodium, Glucose, Insulin

## Abstract

**Introduction:**

Roux-en-Y gastric bypass (RYGB) induces substantial weight loss and changes in glucose homeostasis, partly through alterations in gastrointestinal anatomy and nutrient absorption. Sodium-glucose co-transporters-1 (SGLT-1) facilitate intestinal glucose absorption, a process that may be compromised post-RYGB. Animal studies suggest that sodium supplementation to a meal may enhance SGLT-1 activity post-RYGB. In this study, we investigated whether adding sodium chloride (NaCl) to a carbohydrate-rich meal affects glucose homeostasis in people after RYGB.

**Methods:**

In this open-label, randomized, crossover study, eleven adults without diabetes post-RYGB (mean age 57.3 ± 11.3 years, BMI 35.1 ± 7.5 kg/m2, 64% female) underwent two mixed meal tolerance tests (MMTT), on separate days, either with or without 2 g of additional NaCl (785 mg sodium). The primary outcome was the difference in nadir postprandial plasma glucose between conditions. Secondary outcomes included differences in area under the curve (AUC _0 − 180‘_), fasting and peak plasma glucose, insulin, c-peptide and glucagon-like peptide-1 (GLP-1) concentrations.

**Results:**

The addition of 2 g NaCl did not alter nadir plasma glucose (3.7 mmol/L with NaCl vs 3.5 mmol/L without NaCl, 95% CI: -0.50 to 0.30, p = 0.55), fasting glucose or AUC_0 − 180’_ glucose. However, peak plasma glucose was lower with NaCl (7.8 vs 8.3 mmol/L, 95% CI: -0.88 to -0.08, p = 0.02). No differences were observed in AUC _0 − 180’_, fasting and peak insulin, c-peptide and GLP-1 concentrations between the conditions.

**Conclusions:**

In individuals post-RYGB without diabetes, sodium supplementation during a carbohydrate-rich meal did not substantially affect glucose homeostasis, although it appeared to reduce peak postprandial plasma glucose. Further studies are warranted to explore the clinical relevance of this effect.

**Supplementary Information:**

The online version contains supplementary material available at 10.1007/s11695-025-08420-4.

## Key Points

2 g of NaCl with a meal lowered peak but not nadir postprandial glucose after RYGB.

2 g of NaCl with a meal did not influence postprandial insulin, c-peptide, or GLP-1.

2 g of NaCl with a meal had limited impact on overall glucose homeostasis after RYGB.

**Supplementary Information:**

The online version contains supplementary material available at 10.1007/s11695-025-08420-4.

## Introduction

Roux-en-Y gastric bypass (RYGB) is one of the most commonly performed metabolic procedures worldwide, leading to a sustained mean weight loss of ~ 25% and alterations in glucose homeostasis from the early postoperative period. These metabolic changes often result in remission of type 2 diabetes soon after RYGB [1]. However, in some individuals, they may also lead to adverse outcomes, such as the occurrence of post-bariatric hypoglycaemia (PBH) [2]. While several mechanisms contribute to the changes in glucose homeostasis following RYGB, a key mediator is the modification of the gastrointestinal (GI) tract anatomy, which results in alteration of glucose absorption in the gut.

More specifically, RYGB involves creating a small (~ 30 mL) gastric pouch that is connected directly to a segment of the distal jejunum, forming the alimentary limb. This reroutes ingested food away from the stomach, duodenum, and proximal jejunum - which become part of the biliary limb, carrying bile and pancreatic secretions. The common limb begins at the jejunojejunostomy, where the alimentary and biliary limbs converge, allowing for the mixing of nutrients with digestive secretions which facilitate absorption.

As a result of this anatomical rerouting, ingested food bypasses early contact with bile and pancreatic enzymes, and is rapidly delivered (largely undigested) to the distal small intestine [2–6]. This altered nutrient flow disrupts normal glucose absorption leading to an exaggerated early postprandial rise in plasma glucose and hypersecretion of other key hormones involved in glucose homeostasis, such as insulin and glucagon-like peptide-1 (GLP-1) [2, 6–9]. In some individuals, this response may cause a rapid decline in glucose levels one to three hours after eating, increasing the risk of PBH [7, 9, 10].

Sodium glucose co-transporters-1 (SGLT-1) are important regulators of glucose absorption, driving the majority of glucose uptake in the proximal small intestine [11–13]. In addition to this, SGLT-1s are also thought to contribute to the stimulation of acute GLP-1 release after meals [14]. Due to these important functions, the role of SGLT-1s in glucose homeostasis post-RYGB has been studied further in animals. Baud et al. [15] demonstrated that glucose absorption in post-RYGB mini-pigs primarily occurs in the common limb and is very limited in the alimentary limb. They proposed that the absence of bile (and therefore sodium) in the alimentary limb postoperatively may lead to suboptimal, though functionally intact, SGLT-1 activity, in contrast to the common limb where sodium-rich bile is present [15]. In support of this, when 2 g of sodium were added to the meal of post-RYGB minipigs, the authors observed increased glucose absorption in the alimentary limb and higher postprandial glucose levels [15]. These findings suggest that sodium supplementation may enhance SGLT-1 activity and modify glucose absorption (especially at the alimentary limb) and postprandial glucose levels following RYGB. This should not be interpreted as carbohydrate malabsorption after RYGB, but instead underscores the possibility that luminal sodium availability may influence both the efficiency and anatomical site of glucose absorption within the altered post-surgical anatomy. However, there is a lack of human data evaluating the effect of sodium supplementation on glucose homeostasis after RYGB.

The aim of this study was to investigate the impact of increased dietary sodium intake (2 g sodium chloride, NaCl) taken with a standardized carbohydrate-rich meal on postprandial glucose homeostasis in people without diabetes, following RYGB surgery. We hypothesised that increased sodium intake in humans would enhance luminal sodium availability (particularly in the alimentary limb) and, through effects on SGLT-1 activity, alter the distribution of intestinal glucose absorption by shifting a greater proportion of glucose uptake to the proximal gut. This could reduce the glucose load reaching the distal gut, potentially blunting the exaggerated insulin peak typically observed after RYGB and resulting in altered postprandial glucose levels, including potentially a higher nadir glucose.

## Materials and Methods

### Participants

In this proof of concept, open-label, randomized, crossover study, twelve participants were recruited between April 2021 and January 2024 from primary (GP surgeries, primarily in Leicestershire) and secondary care [metabolic and bariatric centers around Leicestershire (Leicester, Derby and Luton)]. Full inclusion and exclusion criteria can be found in Supplemental file 1. In summary, we included adults who had RYGB surgery at least 12 months prior to study participation. Key exclusion criteria included established diagnosis of PBH, on treatment with medications that can affect glucose homeostasis (e.g. glucose-lowering agents, systemic steroids), a current diagnosis of diabetes and previous revisional metabolic and bariatric surgery (MBS).

### Experimental Procedures/Study Design

**Fig. 1:**
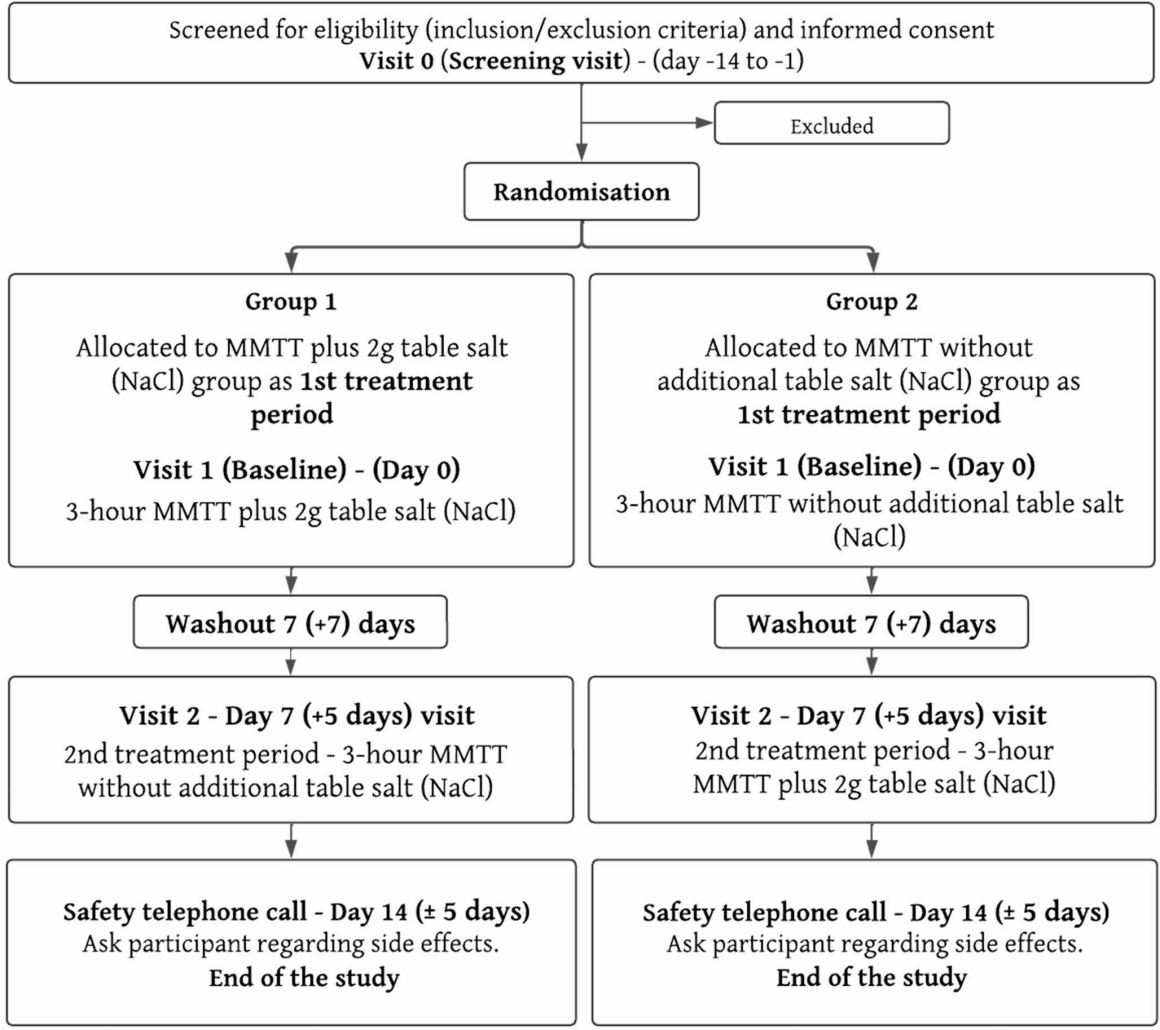
Flowchart of study procedures

All study visits took place at a single research facility (Leicester Diabetes Centre, UK). Figure 1 presents a summary of the study procedures at each visit.

Participants attended a screening visit (Visit 0) to determine their eligibility to take part in the study and to familiarize themselves with study procedures. A general physical examination was performed by a trained delegated clinician. Fasting blood samples were collected to assess HbA1c, full blood count, renal function and liver function. All female participants of child-bearing potential had a urine pregnancy test to confirm their pregnancy status. Demographic and medical history information was also collected.

Following confirmed eligibility, participants attended the first experimental visit (Visit 1). Participants were instructed to fast for ten hours prior to the experimental visits (Visit 1 and Visit 2). For the 24 h before study Visits 1 and 2, participants were asked to refrain from any moderate to vigorous physical activity and alcohol consumption, but were not asked to standardise or record their habitual sodium intake. Anthropometrics [weight, height and body fat percentage (via TANITA bioimpedance scale)] were recorded. Blood pressure and pulse rate were also measured using an automated arm sphygmomanometer whilst participants were seated.

Participants were then randomized (at Visit 1) to one of the two treatment sequences using an online computerized randomization service (sealedenvelope.com). Participants and researchers were informed of the outcome of the randomization. This was due to inability to blind the intervention due to detectable taste of NaCl in the MMTT. Group allocation was counterbalanced to control for order effects. Group one received a standardized mixed meal tolerance test (MMTT) with 2 g of NaCl at Visit 1, and then a MMTT without additional NaCl at Visit 2. Group two received a standardized MMTT without addition of 2 g NaCl at Visit 1, followed by a MMTT with added 2 g NaCl at Visit 2. Two grams of NaCl contain approximately 785 mg of sodium - this amount of NaCl was selected to align with the UK’s recommended maximum daily intake of 6 g [16]. Moreover, this dose was also considered palatable and acceptable, allowing assessment of postprandial responses to a physiologically relevant sodium load consistent with current dietary guidance. During the MMTT, participants were asked to consume 170 mL of smooth orange juice with or without 2 g of NaCl dissolved, followed by a yoghurt snack pot, under researcher supervision. In total, the meal contained 245 kcal, 40 g carbohydrates, 7.5 g protein, 5.5 g of fat and 108 mg of sodium. Participants were asked to consume the standardized MMTT within ten minutes.

An antegrade cannula was inserted into the antecubital vein for blood sampling whilst the participant was seated. Blood samples were collected in the fasted state (immediately before initiation of standardized MMTT) and at 15, 30, 60, 90, 120, 150 and 180 min after MMTT ingestion for measurement of glucose, insulin, c-peptide and total GLP-1 concentrations. A total of 100 mL of blood (approximately 12.5 mL per sample) was collected at Visits 1 and 2. The Sigstad and the Edinburgh Hypoglycemia Symptom Scale (EHSS) questionnaires were completed during the MMTT at the same time points as blood collection to assess dumping and hypoglycemia symptoms respectively [17, 18].

For safety reasons, a bedside blood glucose measurement (using the blood glucose monitoring system Contour Plus Blue, Ascensia Diabetes Care) was also performed at the same time points as blood collection and if glucose levels were ≤ 3.0 mmol/L, independent of symptoms, then the participants would be treated as per the hospital guideline for treatment of hypoglycemia and the MMTT would be stopped. No MMTT had to be stopped due to hypoglycemia in the present study.

After a washout period of at least seven days, participants returned for Visit 2 where they received alternate treatment according to the randomization sequence assigned at Visit 1. All procedures were repeated as per Visit 1 for both groups. Participants received a safety call seven days after Visit 2 to allow them to report any adverse events the week following Visit 2.

### Sample Collection and Biochemical Analysis

Venous blood samples for plasma glucose measurement were collected in 2.7mL fluoride monovettes and were analyzed using a Glucose Hexokinase 3 assay on the Atelica CH (SIEMENS) analyzer by the clinical pathology laboratories of University Hospitals of Leicester NHS Trust. Venous blood for the measurement of insulin and c-peptide was collected in 4.9 mL EDTA (Ethylenediamin tetra-acetic acid) monovettes, while venous blood for GLP-1 analysis was collected in 4.9 mL EDTA monovettes pre-treated with 250 µL aprotinin. Samples for insulin, c-peptide and GLP-1 were spun immediately after collection in a refrigerated centrifuge (4 °C) for 10 min at 1500*g*, before plasma were isolated and stored at -80 °C for subsequent analysis. Plasma insulin and c-peptide were quantified in each sample via a multiplex assay using a Magnetic Luminex Assay (R&D Systems, Minneapolis, MN, USA), and a Luminex LX200 instrument and Xponent software (Luminex Corporation, Austin, TX, USA). Plasma total GLP-1 concentrations were measured using a commercially available enzyme-linked immunosorbent assay (Merck KGaA, Darmstadt, Germany). All samples were assayed in duplicate, with all duplicate samples having a CV% of ≤ 20%. Samples were handled in accordance with the Human Tissue Authority’s Code of Practice.

### Statistical Analysis

Normality of all data was assessed using histograms and the Shapiro-Wilk test, where a p-value greater than 0.05 indicated no significant deviation from a normal distribution. Depending on the distribution of data, participant characteristics were reported as mean (SD) or median (IQR), and number (percentage) for continuous and categorical variables respectively. Descriptive analysis was performed to summarize baseline characteristics of the cohort.

The primary outcome was the difference in nadir (lowest) glucose levels between the two treatments (2 g additional NaCl vs no additional NaCl) after the MMTT. Secondary outcomes included the differences in fasting, area under the curve (AUC_0 − 180’_) and the peak levels of glucose, insulin, c-peptide and GLP-1. Peak was defined as the highest observed postprandial concentration of each parameter for each participant. The trapezoidal rule was used for all AUC calculations to represent total postprandial exposure from time 0 to 180 min. Paired t-test or equivalent non-parametric tests (Wilcoxon signed-rank) were used to analyze the primary and secondary outcomes.

The primary and main secondary outcomes were analyzed on a complete case basis. One participant who completed the study was excluded from the analysis as less than 50% of sample data points were available due to cannulation issues. For the eleven participants included in the analysis, 2% of postprandial data values were missing (14/704) and imputed using a regression method reported previously for acute experimental studies [19–21]. This model used key predictors (age, baseline BMI, fasting values) to derive a regression equation for the glucose, insulin, c-peptide and GLP-1 values at each missing time point. One participant had missing data for all EHSS and Sigstad measures and was excluded, therefore the analysis for EHSS and Sigstad included 10 participants.

For all paired comparisons, differences between conditions were reported with 95% confidence intervals (CI). When both paired variables were normally distributed, a paired t-test was used, and results are presented as the mean difference (95% CI). When either variable was non-normally distributed, the Wilcoxon signed-rank test was applied, given the small sample size, even if the distribution of the differences appeared normal. In these cases, results are presented as the estimated median difference (pseudomedian, 95% CI). The estimated median difference was used instead of the sample median, as it offers a more robust measure of central tendency for non-parametric paired data and aligns with the statistical assumptions of the Wilcoxon signed-rank test.

This was a proof-of-concept study and there were no previous data available on the effect of sodium intake on glucose homeostasis after RYGB in humans. Assuming a standard paired test, the proposed research was powered (80%) to detect a 0.6 mmol/L difference in the primary outcome of nadir (lowest) glucose between the interventions with alpha set at 5% and within person correlation of 0.05 assuming a standard deviation of 0.45 mmol/L. Based on the above assumptions, 11 people were needed to complete the study. A value of *p* < 0.05 was considered statistically significant for all analyses. The analyses were conducted using R software version 4.3.1 (www.R-project.org) and SPSS (version 29.0).

## Results

### Baseline Participant Characteristics

The baseline characteristics of the eleven adults ≥ 12 months post RYGB (mean age 57.3 ± 11.3 years, BMI 35.1 ± 7.5 kg/m^2^, HbA1c 5.4 ± 0.3%, 6.8 ± 5.3 years post-surgery) included in the analysis are presented in Table 1.

**Table 1:** Baseline participant characteristics

Demographic variables (*n* = 11)	
Age (years)	57.3 ± 11.3
Sex	
Male	4 (36)
Female	7 (64)
Ethnicity	
White British	10 (91)
White Irish	1 (9)
Anthropometric variables	
Weight (kg)	98.6 ± 19.0
Height (m)	1.68 ± 0.1
BMI (kg/m^2^)	35.1 ± 7.5
Bodyfat (%)	39.6 ± 10.9
Cardio-metabolic variables	
HbA1c (%)	5.4 ± 0.3
SBP (mmHg)	118 ± 8
DBP (mmHg)	71 ± 7
eGFR (mL/min/1.73m^2^)	86.9 ± 4.7
Years after bariatric surgery	6.8 ± 5.3

*eGFR* estimated Glomerular Filtration Rate, *BMI* Body Mass Index, *SBP* Systolic Blood Pressure, *DBP* Diastolic Blood Pressure, *HbA1c* Glycated Hemoglobin. Data presented as number (%) and mean ± SD

### Glucose

**Fig. 2:**
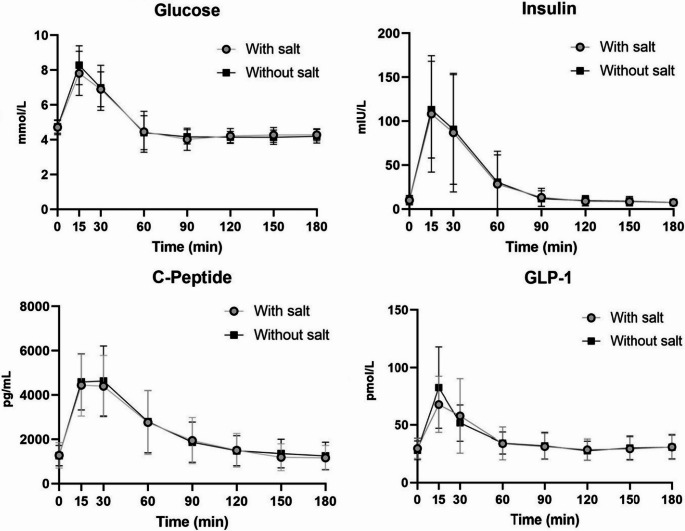
Postprandial plasma concentrations of glucose, insulin, c-peptide and GLP-1 (mean ± SD) during the three-hour MMTT [with and without added table salt (sodium chloride - NaCl)] in 11 participants who have undergone Roux-en-Y gastric bypass

The addition of NaCl did not result in a difference in nadir glucose concentrations compared to standard MMTT [3.70 (3.15–3.95) mmol/L with added NaCl vs 3.50 (3.40–3.80) mmol/L without added NaCl, estimated treatment difference: -0.099 mmol/L, 95% CI: -0.499 to 0.300 mmol/L, *p* = 0.552, Table 2]. NaCl supplementation resulted in 6% lower peak plasma glucose (mean difference − 0.481 mmol/L, 95% CI: -0.880 to -0.083 mmol/L, *p* = 0.022). The overall glucose response (AUC_0 − 180’_) was not different between treatments (*p* = 0.965, Table 2; Fig. 2).

**Table 2:** Fasting and postprandial glucose, insulin, c-peptide, GLP-1, Edinburgh hypoglycemia symptom scale (EHSS) and Sigstad questionnaire responses to an added NaCl (2 g) and no added NaCl mixed meal tolerance test in 11 participants who have undergone Roux-en-Y gastric bypass

Variable	Added NaCl MMTT	No added NaCl MMTT	Mean or estimated median difference (95% CI)	*P*-value
Glucose				
Fasting (mmol/L)	4.71 ± 0.43	4.74 ± 0.37	-0.027 (-0.151 to 0.097)	0.635
Nadir (mmol/L)	3.70 (3.15–3.95)	3.50 (3.40–3.80)	-0.099 (-0.499 to 0.300)*	0.552
Peak (mmol/L)	7.80 ± 1.26	8.29 ± 1.12	-0.481 (-0.880 to -0.083)	**0.022**
AUC_0 − 180’_ (mmol/L*180 min)	841.60 (817.1–966.8)	910.50 (832.1–936.8)	-5.229 (-43.900 to 36.855)*	0.965
Insulin				
Fasting (mIU/L)	7.79 (6.15–12.12)	7.81 (6.85–12.43)	-0.203 (-1.100 to 0.960)*	0.898
Peak (mIU/L)	89.82 (72.34–109.85)	110.44 (95.91–134.28)	-10.81 (-24.96 to 10.23)*	0.320
AUC_0 − 180’_ (mmol/L*180 min)	4483 (3180–5936)	5007 (3693–6307)	-115.8 (-649.69 to 378.55)*	0.577
GLP-1				
Fasting (pmol/L)	29.66 ± 8.76	28.04 ± 8.26	1.622 (-2.177 to 5.421)	0.364
Peak (pmol/L)	69.51 ± 35.20	72.44 ± 29.86	-10.412 (-30.377 to 9.552)	0.272
AUC_0 − 180’_ (mmol/L*180 min)	6705.00 ± 2304.33	6799.00 ± 1972.79	-93.424 (-1127.45 to 940.60)	0.845
C-peptide				
Fasting (pg/mL)	1227.80 ± 572.78	1266.40 ± 464.96	11.481 (-156.267 to 179.230)	0.882
Peak (pg/mL)	4690.00 ± 1376.81	4967.00 ± 1429.50	-277.34 (-637.967 to 83.273)	0.117
AUC_0 − 180’_ (mmol/L*180 min)	414861.00 ± 159780.90	426711.00 ± 156730.20	-11850.13 (-47459.68 to 23759.43)	0.476
EHSS**				
Peak	12.00 (12.00–12.75)	12.00 (12.00–12.00)	-1.485 (-1.50 to 0.50)*^	0.713
AUC_0 − 180’_	2025.00 (1989.00–2044.00)	2025.00 (1999.00–2145)	-60.637 (-157.50 to 26.25)*^	0.529
Sigstad**				
Peak	1.00 (0.00–1.75)	3.00 (0.00–8.00)	-5.999 (-6.50 to 0.00)*^	0.058
AUC_0 − 180’_	22.50 (0.00–63.75)	153.80 (0.00–403.10)	-220.77 (-367.50 to 7.50)*^	0.076

Data is presented as mean ± standard deviation or median (interquartile range), as appropriate. Differences are reported as mean or estimated median difference (pseudomedian) with 95% confidence intervals, based on the test used. P-values were calculated using paired t-test or non-parametric equivalent (Wilcoxon signed rank test). Postprandial concentrations were considered when identifying the peak or nadir value for each participant

*CI* confidence interval, *AUC* area under-the-curve, *GLP-1* glucagon-like peptide-1, *MMTT* Mixed Meal Tolerance Test, *EHSS* Edinburgh Hypoglycemia Symptom Scale, *Sigstad* dumping syndrome symptom questionnaire

*estimated median difference (pseudomedian) with 95% confidence interval

** 10 participants were included in the analysis of EHSS and Sigstad variables (due to 1 participant lacking data for EHSS and Sigstad)

^ 95% confidence interval calculated with a bootstrap resampling method for this parameter

### Insulin

There was no difference in peak plasma insulin concentration or total insulin AUC_0 − 180’_ in response to the NaCl supplemented MMTT and the standard MMTT without added NaCl (*p* = 0.320 and *p* = 0.577 respectively, Table 2; Fig. 2).

### C-peptide

Peak c-peptide levels were similar between the two conditions (*p* = 0.117). Similarly, the overall c-peptide response (AUC_0 − 180’_) did not appear to be influenced by the addition of NaCl in the MMTT when compared to the standard MMTT (*p* = 0.476, Table 2; Fig. 2).

### GLP-1

Peak GLP-1 concentrations and total AUC_0 − 180’_ GLP-1 concentrations did not differ between the NaCl supplemented MMTT and the standard MMTT (*p* = 0.272 and *p* = 0.845 respectively, Table 2; Fig. 2).

### Questionnaire Responses

#### EHSS Score

We observed no difference in the peak or AUC_0 − 180’_ EHSS scores between the MMTT with added NaCl and the MMTT without added NaCl (*p* = 0.713 and *p* = 0.529 respectively, Table 2).

#### Sigstad Score

There were no differences in peak Sigstad score or AUC_0 − 180’_ Sigstad score between the NaCl supplemented MMTT and the standard MMTT without NaCl (*p* = 0.058 and *p* = 0.076, respectively, Table 2).

## Discussion

This is the first study to investigate the acute effect of increased sodium intake with a carbohydrate-rich mixed meal on glucose homeostasis in humans after RYGB. Importantly, this study was not designed to assess sodium supplementation as a therapeutic strategy, but rather to explore whether increased sodium intake (at the dose tested) could influence the postprandial glycaemic regulation after RYGB. We found that the addition of 2 g of NaCl (785 mg of sodium) to a mixed-meal did not increase postprandial nadir plasma glucose levels or alter overall postprandial glucose concentrations, as measured by total AUC. We did however observe a lower peak postprandial plasma glucose concentration with acute NaCl supplementation added to the mixed-meal compared to control, without any differences in postprandial plasma insulin, c-peptide and GLP-1 responses.

There is no comparable data in humans who have undergone metabolic surgery and little evidence is available from animal studies. Although we observed no impact of NaCl on nadir and AUC_0 − 180’_ plasma glucose, we detected a lower peak postprandial plasma glucose concentration. This is contrary to findings from Baud et al. [15] who conducted a study in minipigs and observed higher postprandial plasma glucose excursions (including peak and AUC), along with increased intestinal glucose uptake (assessed via a D-xylose absorption test), following the addition of sodium to a mixed-meal test. The authors attributed this effect to the reintroduction of sodium into the alimentary limb which is typically sodium-deprived due to bile diversion into the common limb after RYGB [15]. Several factors may explain the differences between our findings and those of Baud et al. [15].

Firstly, the composition and method of administration of the test meals differed. In our study, the mixed meal consisted of 170 mL orange juice and a yoghurt pot, providing a total of 245 kcal (40 g carbohydrates, 7.5 g protein and 5.5 g of fat) and was administered orally. In Baud et al. study [15], a meal comprising a 200 mL nutritional supplement and a 20 g solid energy bar (387 kcal, 54 g carbohydrates, 15 g protein and 13 g fat) was administered via a nasogastric tube. Another consideration regarding the mixed meal used is that both the caloric and the carbohydrate load per kilogram of body weight were likely higher in the minipigs than in our participants. Secondly, Baud et al. [15] appeared to have used 2 g of sodium, whereas we used 2 g of NaCl, which contains approximately 785 mg of sodium. The amount of NaCl used in our study was intentionally limited to ensure palatability of the mixed meal and to remain within the recommended daily salt intake guidelines. Finally, it is important to consider the anatomical differences between swine and humans which could have contributed to the disparity in our findings. Although their GI tracts are similar, there are differences including intestinal length (normally longer in minipigs) and rate of gastrointestinal emptying [22–24]. Therefore, it is likely that the outlined differences could limit the reproducibility of the discussed findings from minipigs into human studies.

Whilst no other study has investigated the impact of sodium on glucose homeostasis in people after MBS, studies in non-bariatric populations have been conducted. Findings from Ferrannini et al. [25] show that the addition of 2.7 g of NaCl during a glucose load test (87 g of Glucola), in healthy people who had not undergone RYGB, increased glucose and insulin levels compared to control (glucose load without NaCl). They suggested that NaCl likely enhanced intestinal glucose absorption, as rates of glucose disappearance from plasma were similar between the treatment groups. Whilst this study used a similar design to ours, Ferranini et al. [25] used a liquid glucose solution, which had higher carbohydrate and NaCl content than our mixed meal. In addition, the glucose solution would have quicker absorption in contrast to our mixed-meal, which contained solid/semi-solid elements (yoghurt), proteins and fat, leading to potentially slower absorption [26]. These factors may have also contributed to the disparity between our results, in addition to the lower sodium content in our study and the differences in digestion between people who had undergone RYGB and those who had not. These considerations highlight the need for further studies to better understand sodium–carbohydrate interactions after RYGB.

Based on our observation of lower peak plasma glucose levels following the mixed meal, we might have expected a corresponding reduction in peak plasma insulin levels. However, we did not detect any significant changes in overall or peak insulin, c-peptide, or GLP-1 levels in response to the meal’s increased sodium content. This suggests that the reduction in peak plasma glucose may be driven by alternative mechanisms, such as enhanced insulin sensitivity, similar to findings in studies with high-salt vs. low-salt diets in non-bariatric populations [27, 28]. Our observation of lower peak postprandial glucose is difficult to reconcile and could also be the result of a type 1 error; therefore further investigation, including studies using stable isotope tracers, would help to confirm this observation and explore potential underlying mechanisms.

While our study aimed to enhance SGLT-1 activity at the gut via sodium chloride provision, our findings suggest this approach may not significantly modulate overall glucose levels or hormone responses, at least at the tested dose. A recent study by Martinussen et al. [29] investigated the opposite approach - inhibition of SGLT-1 using canagliflozin 600 mg (a dual SGLT-1/SGLT-2 inhibitor) in people after RYGB. They found delayed glucose absorption and attenuated early postprandial GLP-1, glucose-dependent insulinotropic polypeptide (GIP), glucose and insulin responses, suggesting that SGLT-1-mediated glucose uptake contribute to the rapid hormone release postoperatively. Together, these studies highlight the need to better understand the role of SGLT-1 in regulating glucose homeostasis and insulin secretion in humans following RYGB.

The key strength of this study is the randomized, cross-over design, the novelty of the intervention and the detailed assessment of glucose, insulin, c-peptide and GLP-1 concentrations through a standardized MMTT. We did not investigate mechanisms of glucose absorption in this study, and our static measures of plasma glucose concentration meant that we were not able to distinguish between the appearance and disappearance of plasma glucose and sodium’s mode of action. Additionally, we did not control or quantify habitual sodium intake in our participants prior to the experimental visits, which may have affected the physiological responses to acute sodium supplementation. Recording or standardizing baseline sodium intake in future studies may help reduce this potential variability. Although we observed a reduction in peak postprandial plasma glucose levels, suggesting a potential effect of sodium on glucose homeostasis, the underlying mechanisms are difficult to discern due to the discussed limitations and this may have resulted from a type 1 error. We used a MMTT instead of an oral glucose load because it provides a more physiological stimulus and better reflects real-world nutrient intake. Importantly, it also reduces the risk of triggering early dumping symptoms, which are common after RYGB and can be provoked by high-glucose loads. The mixed meal deliberately contained only 40 g of carbohydrates to further minimise this risk which may have limited the postprandial rise in glucose levels and the subsequent effects on glucose homeostasis, including the secretion of insulin, c-peptide and GLP-1. Additionally, the 2 g NaCl dose, selected for palatability, may have been insufficient to elicit a more robust physiological response. This amount represents approximately 24% of the average daily NaCl intake in the UK (8.4 g/day, based on urinary sodium excretion data) and 33% of the UK recommended upper limit of 6 g/day [30]. While higher doses of NaCl with a single meal might have produced a more pronounced effect, further supplementation in our study was deemed neither practical (mainly due to palatability concerns with the test meal), nor consistent with the current UK guidance to limit NaCl intake to no more than 6 g per day [16]. Furthermore, the impact of the COVID-19 pandemic on surgical activity and recruitment timelines may have affected the generalizability of the findings to the broader post-RYGB population.

In conclusion, the addition of 2 g NaCl to a carbohydrate-rich meal decreased peak postprandial glucose levels but did not affect nadir or overall postprandial plasma glucose levels in post-RYGB patients without diabetes or a diagnosis of PBH. This exploratory study was not designed to evaluate sodium as a therapeutic intervention, but rather to assess whether acute sodium supplementation (at the tested dose) can influence postprandial glucose regulation. Future studies could examine the potential effects of higher sodium doses, different sodium-to-carbohydrate ratios, or prolonged sodium supplementation on glucose homeostasis after RYGB, as well as the effects of these interventions in individuals with PBH. Determining whether such effects are clinically meaningful, and whether threshold effects exist, could ultimately inform nutritional guidance for individuals following RYGB.

## Supplementary Information

Below is the link to the electronic supplementary material.

Supplementary Material 1

## Data Availability

Data included in this manuscript is available upon request from the corresponding author.
