# Supplementary material for: The Effect of Increased Sodium Intake with a Carbohydrate-Rich Meal on Glucose Homeostasis in People without Diabetes after Roux-en-Y Gastric Bypass: a Proof-of-Concept, Randomized, Open-Label, Crossover Study
Source: Obes Surg. 2025 Nov 28;36(1):215–25. doi: 10.1007/s11695-025-08420-4 (PMC12852143; doi:10.1007/s11695-025-08420-4)
Supplement: Supplementary file 1 — Supplementary Material 1 [file 11695_2025_8420_MOESM1_ESM.docx]

Full Inclusion/exclusion criteria (sourced from study protocol v3.8 17.11.2023)

**Inclusion Criteria:**

• Aged≥18 years old but less than 75 years old

• Subjects ≥1 year after gastric bypass (RYGB)

• Able to understand written and spoken English

• Able to give informed consent

**Exclusion criteria:**

• Use of any glucose-lowering medication (including insulin)

• Adrenal insufficiency and/or substitution with glucocorticoids

• eGFR≤45 ml/min/1.73m^2^

• Recent active infection (an active infection will be any infection over the last 10 days)

• Current use or history of treatment within 6 weeks with systemic glucocorticoids (oral or injectable) not including use of topical (e.g. eye drops or topical creams) or inhaled glucocorticoids.

• Known primary or secondary hyperaldosteronism

• Severe hypertension during screening visit (Systolic Blood Pressure >180mmHg as average of 3 measurements)

• Established diagnosis of congestive heart failure

• Significant peripheral oedema on clinical examination at screening visit

• People with allergies (e.g. milk protein allergy) or severe intolerance to the mixed meal test as assessed by the clinician (e.g. severe lactose and gluten intolerance)

• People following a vegan diet (mixed meal tolerance test not suitable for those following a vegan diet)

- Other bariatric procedures except for RYGB or gastric band which have been removed

• Previous revisional bariatric surgery (except for gastric band which has been removed)

• Currently pregnant or breastfeeding

• Diagnosis of type 1 diabetes

• Current diagnosis of type 2 diabetes (defined as HbA1C ≥6.5% at screening blood tests or HbA1C <6.5% at screening bloods but on glucose lowering medications over the last 3 months)

• Patients with diagnosis of Epilepsy

• HbA1C ≥6.5% or ≥48 mmol/l at screening blood tests

• Hemoglobin (Hb) <100 g/L at screening blood tests

• Participating in a Clinical Trial of Investigational Medicinal Product (CTIMP) within <1 month of screening.

• Having a formal previous diagnosis of postprandial hypoglycemia

• Being on acarbose, diazoxide, octreotide or other treatment for postprandial hypoglycemia

• Any concurrent condition, in the judgment of investigator and/or sponsor, that could interfere with the safety and study conduct or interpretation of study results
